# 2428 Obstructive sleep apnea as an independent predictor of postoperative delirium and pain: An observational study of a surgical cohort

**DOI:** 10.1017/cts.2018.173

**Published:** 2018-11-21

**Authors:** Patricia K. Strutz, Vanessa L. Kronzer, Mark D. Willingham, Amrita Aranake-Chrisinger, William S. Tzeng, Arbi Ben Abdallah, Simon Haroutounian, Michael S. Avidan

**Affiliations:** Institute of Clinical and Translational Sciences, Washington University in St. Louis

## Abstract

OBJECTIVES/SPECIFIC AIMS: To study the role of OSA as an independent predictor of perioperative outcomes. METHODS/STUDY POPULATION: For this single-institution cohort study, we included data from patients who were enrolled into 1 of 3 prospective parent studies. All participants underwent in-patient surgeries, excluding neurosurgeries, which required general anesthesia and a postoperative stay of at least 1 day. Patients included in this study were assessed daily for postoperative delirium and pain severity as part of the parent studies. In the current study, determination of delirium diagnosis was based on the 3-minute Diagnostic Confusion Assessment Method (3D-CAM), and the Visual Analogue Pain Scale (VAS) was used for pain severity. Data on OSA diagnosis (determined by sleep study); OSA risk (determined by the STOP-Bang tool; snoring, tiredness, observed apnea, high blood pressure, body mass index>35 kg/m^2^, age>50, neck circumference, male gender); and compliance with treatment were obtained from the preoperative assessment record. Participants were grouped into 1 of 3 categories: high risk of OSA (HR-OSA; including patients with a previous positive sleep study or STOP-Bang score ≥5); intermediate risk of OSA (IR-OSA; including patients with a STOP-Bang score of 3 or 4); and low risk of OSA (LR-OSA; including patients with a previous negative sleep study or STOP-Bang score <3). Candidate risk factors for delirium and pain were also extracted from this record. RESULTS/ANTICIPATED RESULTS: Logistic regression will be used to test whether OSA independently predicts postoperative delirium and linear regression to assess OSAs relationship to acute pain severity. We hypothesize that patients in the HR-OSA category will experience a higher incidence of postoperative delirium and greater postoperative pain severity. We also predict a step-wise increase in risk of these adverse outcomes when analyzing patients stratified by OSA risk (HR-OSA vs. IR-OSA vs. LR-OSA). For our secondary analyses, we anticipate these outcomes are modified by compliance with CPAP treatment. We believe patients with OSA who do not use prescribed CPAP will experience a higher incidence of postoperative delirium as well as increased pain severity. DISCUSSION/SIGNIFICANCE OF IMPACT: OSA is a common and frequently undiagnosed perioperative problem associated with altered pain processing and a high incidence of postoperative delirium. While likely providing stronger evidence of OSA’s reported impact on postoperative delirium and pain, our findings might also help discern points of intervention for treatment and prevention. Since OSA’s presumed impact poses challenges to clinicians and patients, prospective, randomized trials testing preventative or mitigating interventions are necessary. We hope to use these results to design such trials and clinical plans, with the goal of reducing postoperative delirium and acute postsurgical pain severity for the large number of patients at risk due to OSA.

